# Effect of a pre-exercise energy drink (Redline®) on upper-body muscular endurance performance

**DOI:** 10.1186/1550-2783-8-S1-P18

**Published:** 2011-11-07

**Authors:** Jay Dawes, Liette B Ocker, David R Temple, Frank Spaniol, Alison M Murray, Randy Bonnette

**Affiliations:** 1 Department of Kinesiology. Texas A & M University-Corpus Christi, Corpus Christi, TX, USA

## Background

Energy supplements are frequently consumed by athletes and recreational fitness enthusiasts as a method of improving exercise performance. Recent research indicates that these types of supplements influence exercise performance by increasing the number of repetitions that can be performed during an acute bout of exercise, thus increasing the total volume of work that can be performed during training sessions (Hoffman et al., 2008). Therefore, when aiming to improve muscular endurance performance the use of such a supplement may enhance one’s ability to withstand fatigue. The purpose of this study was to investigate the effect of a high energy liquid supplement on upper-body muscular endurance performance.

## Methods

Forty-one healthy males (21.73 ± 1.74 yrs; 176.48 ± 7.54 cm; 81.16 ± 10.94 kg) volunteered to participate in this study. All test subjects completed a health history and caffeine usage questionnaire, as well as an informed consent form, prior to participating. Subjects completed a pre and post push-up to fatigue test within a week of one another. During the post-test session subjects were either given four ounces of an energy supplement (Redline by VPX) or a placebo, 30 minutes prior to the push-up to fatigue test. Administration of the supplement was double blind. Twenty-three (n=23) subjects received the supplement, while eighteen (n=18) subjects received the placebo. A 2 x 2 factorial ANOVA was used to determine between group differences for the muscular endurance assessment, at an alpha level of 0.05.

## Results

Data analysis revealed a significant interaction between the treatment effect and the trials, *F* (1, 40) = 4.13, *p* = 0.024. Moreover, no significant difference was found between the pretest treatment group and the pretest placebo group, *F* (1, 40) = 3.07, *p* = 0.09, indicating that all subjects began the study with similar upper-body muscular endurance. Further examination of posttest main effects revealed a significant difference between the treatment group and the placebo group, *F* (1, 40) = 6.99, *p* = 0.01. The pretest push-up scores were similar for the treatment (52.91 ± 18.93) and the placebo group (44.22 ± 10.28). However, the treatment group showed substantially greater push-up scores for the posttest (59.34 ± 19.58) than the placebo group (45.66 ± 11.16). This represented a 12.15% increase in the treatment group’s posttest scores and a 3.25% increase in the placebo group’s posttest scores.

## Conclusions

The results of this study indicate that the pre-exercise, liquid energy drink energy supplement investigated in this research had a significant effect on upper-body muscular endurance as measured by the push-up to fatigue test.

